# Nail-Gun Injuries to the Hand

**Published:** 2008-11-13

**Authors:** Yvonne N. Pierpont, Effie Pappas-Politis, Deepak K. Naidu, R. Emerick Salas, Erika L. Johnson, Wyatt G. Payne

**Affiliations:** Institute for Tissue Regeneration, Repair, and Rehabilitation, Bay Pines VA Healthcare System, Bay Pines, Florida, and Division of Plastic and Reconstructive Surgery, University of South Florida, Tampa

## Abstract

**Background:** The nail gun is a commonly utilized tool in carpentry and construction. When used properly with appropriate safety precautions, it can facilitate production and boost efficiency; however, this powerful tool also has the potential to cause serious injury. The most common site of nail-gun injuries in both industrial and nonoccupational settings is the hand. **Materials and Methods:** We report on two patients with nail-gun injuries to the hand. A review of the literature and discussion of clinical evaluation and treatment of nail-gun injuries to the hand are presented. **Results:** Two patients present with soft tissue injuries to the hand with the nail embedded and intact at the injury site. Operative removal of the nail and wound care resulted in successful treatment in both cases. Nail-gun injuries to the hand vary in severity on the basis of the extent of structural damage. Treatment is based on the severity of injury and the presence and location of barbs on the penetrating nail. **Conclusion:** Healthcare providers must understand and educate patients on the prevention mechanics of nail-gun injuries. Nail-gun injuries to the hand necessitate appropriate evaluation techniques, understanding of surgical management versus nonsurgical management, and awareness of potential pitfalls in treatment.

Pneumatic nail guns are efficient, readily available, and easy to use, making them a common tool employed in residential construction and wood-production industries.^1^ In addition, nail guns are frequently utilized by the nonprofessional consumer population for general construction. The nail gun is a mechanical device used to frame wooden structures, secure wood to concrete supports, and in multiple other construction and home improvement applications.^2^ Powered by either an explosive charge or compressed air, this tool generates enough force to fire a projectile up to 10 cm in length, with velocities as high as 1400 feet per second, into fully stressed concrete.^3^–^5^ This can be equated to the firing capacity of a .22 caliber handgun or rifle.^2^,^3^,^6^,^7^ High-velocity devices eject nails by detonating explosive cartridges directly behind the gun barrel.^5^,^8^–^10^ The more commonly used lower velocity nail guns, in comparison, eject nails indirectly by activating a captive piston usually by means of compressed air. In both devices energy is utilized to fire bolts, metal studs, nails, pins, and fasteners into wood, metal, concrete, and masonry by pressing a contact trip or sequential triggering mechanism on the gun.^3^,^7^,^11^

Since the introduction of pneumatic-powered nail guns to the construction industry in 1959, there has been an increasing number of industrial accidents involving these devices, with the most frequent area of injury being the hand.^5^,^12^–^14^ The radial aspect of the nondominant hand, as it is typically used to grip or steady the structures being nailed, can easily cross the nail's line of fire, and thereby is the body part most often injured.^2^,^12^–^20^ Most of these workplace injuries occur during routine use and are due to accidental discharge, careless handling of equipment, overpenetration of structures by the projectile, ricochet or shattering of the projectile, and the structural unsoundness of the receiving material.^7^,^11^ The majority of injuries involve retained nails with trauma limited to the surrounding soft tissues. Direct bony injuries to the digits, hand, and wrists as well as penetrating injuries to the interphalangeal and radiocarpal joints have been described.^5^,^13^,^14^,^19^ Consequently, a systematic approach toward understanding the mechanisms of nail-gun injury, as well as recognizing complicating factors in the surgical management of these injuries, is essential for appropriately treating these patients. We present two cases of nail-gun injuries to the hand and outcomes of nail removal.

## CASE REPORT 1

**Figure 1:**
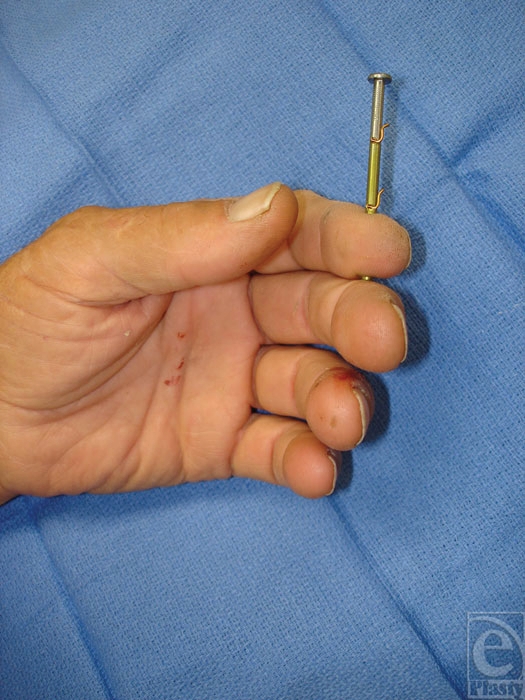
Nail gun framing nail injury with barbs located outside the soft tissue of the fingers.

**Figure 2:**
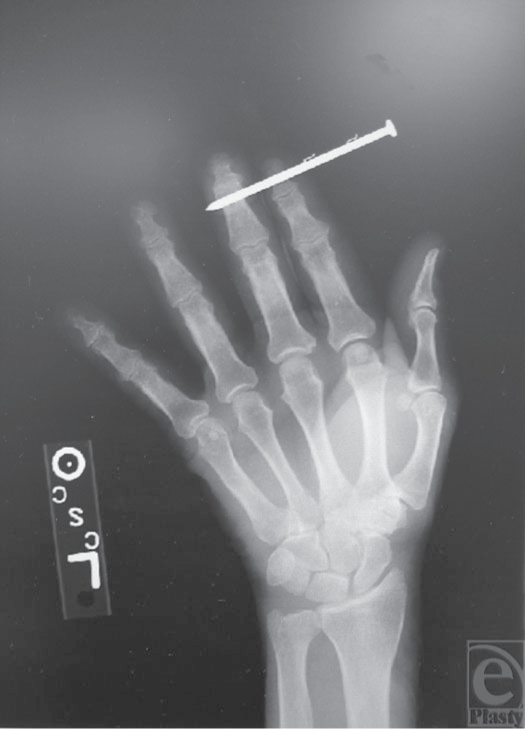
X-ray film demonstrates no bony injury with barbs located outside the soft tissue of the fingers.

A 65-year-old right-handed male construction worker inadvertently fired an air-powered nail gun into his left index and middle fingers (Fig 1). Visual inspection revealed that the nail was penetrating the volar soft tissue through and through the distal phalanges of the index and middle fingers. The patient complained of pain, but there were no apparent motor, neurologic, or vascular deficits. Although the range of motion was limited because of retained nail, there was no indication of tendonous injury. Two sharp barbs were protruding from the nail. The patient thought there was a third barb imbedded in his skin, but it was not observed by examination. X-ray imaging showed no evidence of associated fracture and displayed the two barbs seen on physical examination proximal to the injury site (close to the nail head) with no evidence of barbs within or distal to theinjury site (Fig 2).

The nail was removed in the operating room. It was slowly manipulated and removed in a retrograde fashion without any obvious complications. Fluoroscopic x-ray film verified that no retained foreign bodies remained in the wound. Thorough irrigation was performed with pulse lavage. Topical antimicrobials were utilized for wound care as an outpatient. The patient's recovery was uneventful with no residual functional deficit.

## CASE REPORT 2

**Figure 3:**
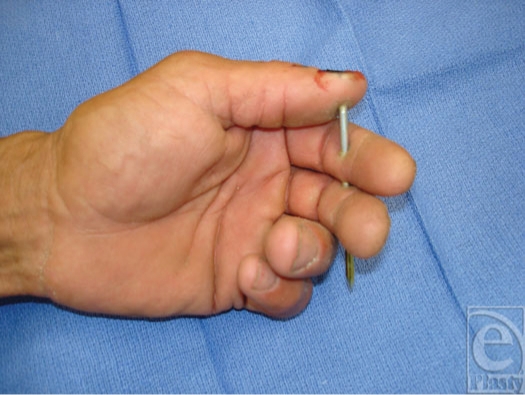
Nail gun framing nail injury involving thumb, index, and middle fingers.

**Figure 4:**
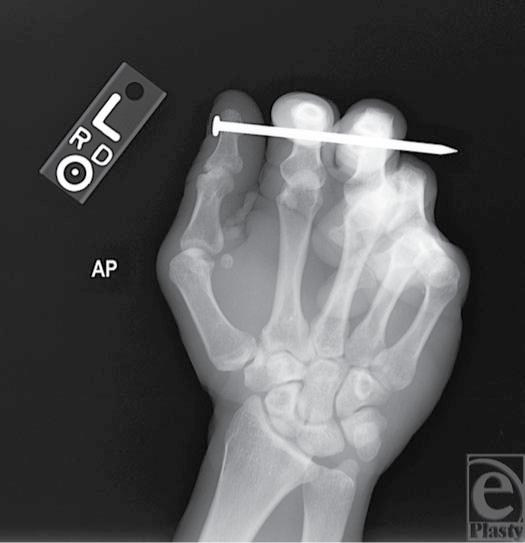
X-ray film demonstrates no bony injury to phalanges (AP [anteroposterior] view).

**Figure 5:**
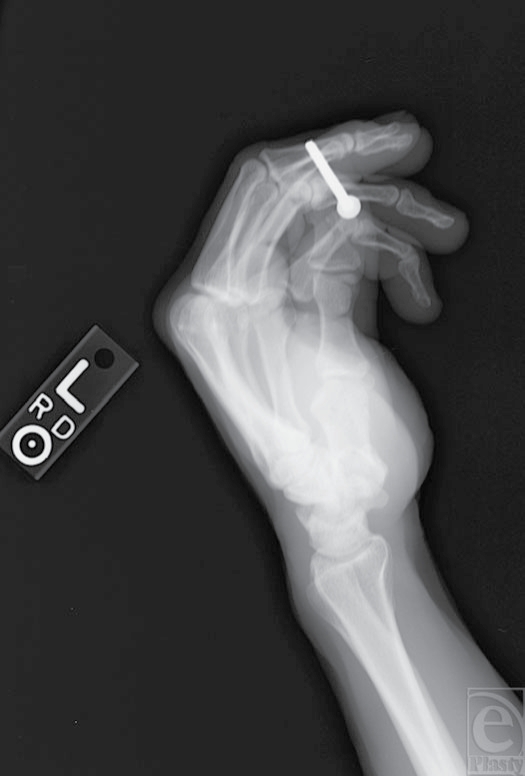
X-ray film demonstrates no bony injury to phalanges (oblique view).

A 44-year-old right-handed male construction worker presented to the emergency department with a nail deeply embedded in his left thumb, index, and middle fingers. While house framing, he had fired an air-powered nail gun into his nondominant hand (Fig 3). Pain and the retained framing nail limited the ability to assess functional status of the injured digits; however, neurologic and vascular function appeared intact. Plain film x-rays did not reveal any evidence of fracture (Figs 4 and 5).

**Figure 6:**
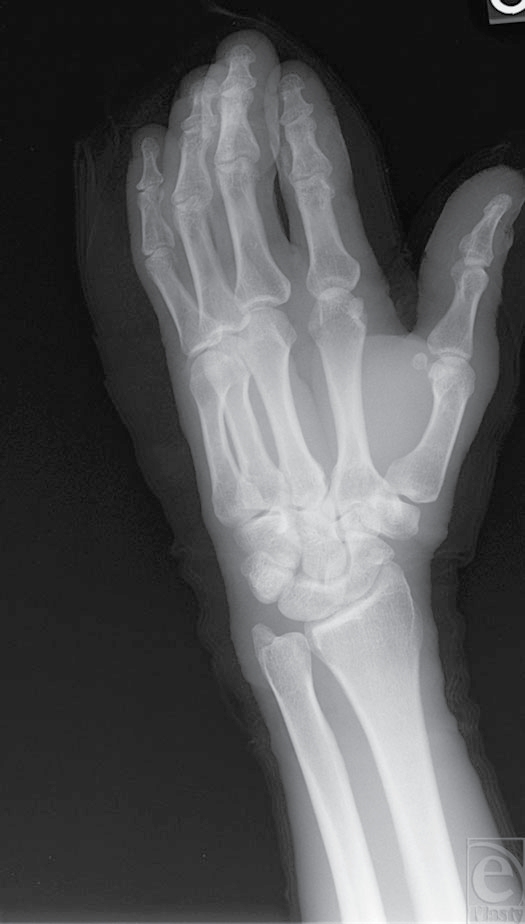
Postextraction x-ray film demonstrates no bony injury.

The nail was removed in the operating room by retrograde extraction. There appeared to be no tendonous or bony injury. All puncture wounds were thoroughly irrigated and treated open with topical antimicrobial dressing. Immediate postoperative plain-film x-ray confirmed the absence of bony injury (Fig 6). Daily wound care was prescribed, and the wounds healed rapidly and the patient had no functional impairment.

## DISCUSSION

The hand is a commonly injured area of the body, with 16% to 22% of all hand injuries occurring at work.^13^,^21^ As the average house construction requires 50,000 to 70,000 nails, nail gun use can significantly increase the speed of construction.^10^,^22^ Increased productivity, however, comes with an increasing number of injuries associated with the device.^10^ In residential carpentry, nail-gun injuries account for 14% of injuries, with more than half of these involving penetrating injuries to the hand or fingers.^17^ Dement et al found that nail-gun injuries were responsible for 3.9% of workers' compensation claims in North Carolina and Ohio, with a majority comprising injuries of the hands and fingers (55%‐57%).^22^ Similarly, compensation claims from Washington state estimated the rate of nail-gun injuries in wood frame construction to be 2.06 per 200,000 hours worked, 66% of these injuries involving hands and fingers.^17^,^23^,^24^ Not surprisingly, injuries may also be underreported because workers often seek medical attention only when the injury is deep or when the nail cannot be easily removed.^13^

Injuries associated with the use of compression guns vary widely in site and severity.^8^,^18^,^25^–^28^ The amount of energy required to cause serious injury is fairly low: penetration of the skin occurs with projectile velocities of 150 feet per second, whereas bony fractures may occur with projectile velocities of 195 feet per second.^7^,^29^ In comparison, the velocity of the projectiles fired from nail guns can reach up to 1400 feet per second, leading to extensive damage when injury occurs. Although the most common site of injury in industrial and nonoccupational settings is the hand, case reports have described injuries to the thorax, abdominal wall, flank, pelvic wall, facial bones, and skull.^2^,^17^,^26^,^30^–^32^ Paralytic spinal cord transection, bowel perforation, long bone fracture, liver laceration, hemopneumothorax, blindness, cerebral damage, and even fatal injuries have been reported.^2^–^4^,^11^,^17^,^25^,^28^,^30^–^39^

When nail projectiles penetrate human tissues, the kinetic energy transfers from the object to the surrounding tissues, resulting in shock waves that form temporary and permanent cavity spaces.^7^,^29^,^39^,^40^ As these shock waves expand, the temporary cavity created causes crush and stretch damage to tissues.^7^,^39^,^40^ If the path of the projectile is influenced by yaw, tumble, or ricochet from surrounding structures, this will generally widen the extent of tissue injury. If the projectile shatters bone, these fragments act as secondary missiles, further increasing tissue trauma.^7^,^41^

Injuries are often further complicated by contamination with skin, oil, paper, or glue.^42^ Nails are held together with wire or paper and adhesive, which can be drawn into the wound with the nail.^10^ The head of the nail itself can lacerate a small portion of skin and/or clothing as it drives into the body leaving this material deep in the wound. The combination of tissue edema, devitalized tissue, and foreign matter provides an ideal environment for local infection.^2^,^7^,^43^

Hand injuries can be classified as direct bony injury, injury to joint, tendon, or nerve, and isolated soft tissue injury.^42^ Despite the intricate and complex anatomy of the hand, the majority of all nail-gun injuries result in isolated soft tissue damage only. One series reported that only 25% of nail-gun injuries to the hand resulted in structural damage, including fracture, longitudinal tendon split or puncture (no division), joint capsule penetration, and bruised digital nerves or neurapraxia (without division of the nerve). Although neurovascular injury is uncommon with nail-gun injuries to the hand, the nail may be in close proximity to a neurovascular structure.^5^,^13^ Consequently, poor understanding of the injury or failure to recognize the mechanism of injury can lead to iatrogenic injury during treatment.^5^

Of particular concern for iatrogenic injury is the presence of “barbs” on the imbedded nail.^14^ Copper wire fragments join nails into strips, which are preloaded into the nail gun. As the nail exits, pieces of copper wire can shear off the strip and remain attached to the nail, creating a sharp, protruding “barb,” which may be a possible source of further injury or complicate the extraction of the nail.^5^,^14^ Open exploration and extraction of the barb under direct vision will help avoid secondary damage.

Evaluation of the patient with a retained nail in the hand begins with a careful history and physical examination. Special attention should be given to the type of nail gun used, the mechanism of injury, and the amount of time elapsed since injury onset. Status of tetanus immunization must also be determined and appropriate treatment should be given. Physical examination should note the general appearance of the hand, obvious fractures or deformity, limitations on range of motion, and proximity to important structures. Capillary refill and two-point discrimination is needed to assess neurovascular status. Absent pulses, an insensate digit, suspected joint penetration, fracture, or tendon injuries require immediate surgical consultation and operative exploration. Radiographs with a minimum of two views of the hand should always be obtained: the films must be scrutinized for associated fractures, joint penetration, and the presence of metallic barbs on the nail shaft.

Treatment of a retained nail following nail-gun injury adheres to standard principles of wound management. A single dose of intravenous or intramuscular antibiotics is often administered, usually a first-generation cephalosporin to cover skin flora contamination in uncomplicated cases. Regional blocks are usually sufficient for wound exploration and debridement. The wound and surrounding soft tissues are thoroughly irrigated with isotonic sodium chloride solution with or without the addition of antimicrobial agents. Exploration for removal of foreign material and to avoid secondary injury from nail extraction (barbs are not always visible on x-ray film) should be considered.^2^,^14^,^42^

A series of 88 nail-gun injuries to the extremities noted a low frequency of infection and rapid return of function with patients who underwent nonsurgical nail removal.^10^ This suggests that nail-gun injuries to the extremities, when there is no articular or neurovascular involvement, can be managed with simple extraction, minimal debridement, and a short course of oral antibiotics. Specific suggestions in appropriate cases for nonoperative extraction recommend removing the head of the nail at the level of the entrance wound and withdrawing the nail slowly, in an antegrade direction, through the exit wound.^2^,^15^,^20^

In cases with suspicion of injury to the joint space, tendons, or neurovascular bundles, intraoperative exploration is indicated.^5^ Cautious nail removal with adequate wound debridement of crushed or devitalized tissue, removal of foreign material, irrigation, and open wound drainage with preoperative and postoperative antibiotic coverage is necessary.^5^,^19^ Wounds may be left open or closed dependent upon the extent and nature of the injury and the level of contamination.^5^ Close follow-up within 48 to 72 hours postoperatively in uncomplicated cases is advised, while inpatient observation may be indicated in cases of a more significant nature.

Most accidents involving nail guns result from operator inexperience, lack of knowledge, inattention to safety precautions, or poor mechanical safety mechanisms of the nail guns.^17^,^22^,^44^ Injuries occur from various circumstances, including nail ricochet, gun double firing, accidental discharges, and penetration of the receiving structure.^3^,^17^,^22^,^38^,^44^ Nail guns should be used only by knowledgeable, educated, and experienced personnel, with proper protective clothing, and precautionary measures should be clearly displayed at all times.^36^ Unfortunately, the incidence of industrial hand injuries remains high despite advances in health and safety awareness. Some studies have questioned the adequacy of on-the-job training and suggested more extensive operator training and improvement in protective clothing.^13^,^45^

Revision nail-gun safety mechanisms and use of newer sequential triggers may prevent accidental misfires. The older and more commonly used contact trip trigger nail guns allow nails to discharge from the tool anytime the nose and trigger mechanism are both depressed.^13^,^17^,^22^,^44^ Accordingly, the operator may keep the trigger depressed during rapid fire “bounce” nailing and may accidentally contact the steadying hand or other body part in lieu of the structure itself. Sequential trigger nail guns, on the other hand, require the nose of the nail gun to be depressed first—before the trigger is pressed—to fire a nail, which makes it more difficult to unintentionally discharge nails. Lipscomb et al studied 772 apprentice carpenters, carpenters with four or less years of experience, finding that approximately half of these carpenters would sustain a nail-gun injury before they completed their 4-year apprenticeship training.^44^ It was also not uncommon for them to be injured more than once by a nail gun during this period. Exposure to tools with contact trip trigger mechanisms carried twice the risk of injury than did tools with sequential triggers after adjusting for training and experience.^44^,^45^ Of the injuries noted, more than 40% of the contact trip injuries occurred when the carpenter was “bounce” or “bump” nailing. Although contact trigger use may not be the sole contributing factor, an increased likelihood of injury due to reduced control of accuracy is inevitable.

Hand injuries represent a significant cause of disability and decreased productivity in occupational and nonoccupational settings. In the case of nail guns, healthcare providers must understand and educate patients on the prevention mechanics of nail-gun injuries, be aware of the appropriate evaluation and indications for surgical management versus nonsurgical management, and recognize potential pitfalls in treatment choice.
